# Editorial: Women in crop physiology and derived products: 2022

**DOI:** 10.3389/fpls.2023.1167837

**Published:** 2023-04-04

**Authors:** Petronia Carillo

**Affiliations:** Department of Environmental, Biological and Pharmaceutical Sciences and Technologies, University of Campania “Luigi Vanvitelli”, Caserta, Italy

**Keywords:** gender equality, sustainable agriculture, circular economy, 2030 agenda, gender gap

Women obtain approximately 50% of all European university doctoral graduations, but they are under-represented in research careers, being approximately 30% of all researchers in Europe and globally and only 12% in the academic sector (UIS, 2019; OEDC, 2021). The gender gap in research has even widened due to the pandemic, disproportionately affecting female researchers even though some have been at the forefront of the crisis response. In fact, women have had to reduce the time dedicated to research more than men to take care of the family and children, having greater difficulty in keeping their jobs or in having career opportunities (UNESCO, 2021). This Research Topic in Frontiers in Plant Science was promoted to empower and encourage the work of female scientists who carry out research in all fields of crop physiology and derived products. Their work is pivotal for achieving the 2030 Agenda for Sustainable Development. Extreme environmental conditions have, recently, led to an intensification of the extent and frequency of stress phenomenon in plants, significantly reducing world agricultural production. According to the Intergovernmental Panel on Climate Change (IPCC), human activities are the main factor responsible for climate instability, with agriculture contributing approximately 50% of total CH4 and N2O anthropogenic emissions (IPCC, 2018). Female scientists can give a great contribution to the transition towards more sustainable models of agricultural production, being less tied to the stereotypes between conventional and alternative agriculture and being more open to innovation. The Mediterranean Basin and the Middle East, already characterized by dry and hot climates and persistent rain deficits, are considered hot spots for climate change due to the growing occurrence of a decrease in precipitation of up to 30% and predicted average warming of 2 to 5°C by the end of the 21st century (IPCC, 2021). In the view that increasing plant diversity in agricultural systems may help sustainably increase crop yields even under drought, Singh et al. studied the effect of drought on bean production mediated by intraspecific variations in crop mixtures. A greenhouse experiment, with and without drought treatment, was carried out with the three selected bean varieties more tolerant to drought and were differently combined with sunflower, chickpea, and sorghum as companion plant species (crop mixtures, bean cultivar mixtures, and monocultures). The findings showed that the effect of companion plant species was dependent on the bean variety but not influenced by drought stress; whereas, the chickpea drought tolerance potential and yield were higher in plant mixtures than in its monoculture. Therefore, the authors demonstrated that for developing drought-tolerant plant mixtures, both the functional traits of the interacting plant species and the different plant varieties must be considered. Melatonin (MEL; N-acetyl-5-methoxy tryptamine), a phytohormone-like molecule, may play a role in modulating plant growth and endogenous plant tolerance mechanisms against abiotic stresses, including drought and heavy metal toxicity. Accordingly, Mir et al. studied the effects of MEL in mitigating the deleterious effects of (40 μM) Cu pollution on morpho-physiological and biochemical parameters in Brassica juncea. They found that MEL was able to increase the nutrients use efficiency and decrease Cu-dependent oxidative stress, ameliorating the stability of chloroplast and stomata. This allowed for the improvement of mustard photosynthetic efficiency and growth, therefore, not only promoting resilience to Cu stress but also boosting the ability of the MEL-treated plants to increase the remediation of Cu-contaminated soils. The use of synthetic herbicides for weed control has also had a strong negative impact on the environment and biological diversity due to the accumulation of these toxic chemicals in the soil and water. Colbach et al. performed a simulation study for identifying ideal pea varieties and new cropping tools for performing agroecological weed management. They started parameterizing the FLORSYS model with contrasting pea varieties by using data from experiments and literature and running virtual experiments with the best pea varieties in different cropping systems to identify the pea parameters making peas competitive against weeds. This allowed for the creation of new rules to help farmers identify the best pea variety, depending on the cropping system and production goal, bridging the gap between minimizing yield losses due to the presence of weeds and increasing/maintaining yield potential. In order to address the increasing food demand, Lu et al. summarized the recent biotechnology and breeding studies with the aim of increasing grain number per panicle (GN), which is the most effective way to improve rice grain yield. They suggested that gene editing technologies, among which are clustered regularly interspaced short palindromic repeats (CRISPR-Cas9), transcription activator-like effector nucleases (TALENs), and zinc-finger nucleases (ZFNs), can be useful tools for modulating crop breeding and increasing GN. Moreover, given the pleiotropy of GN-associated genes, they explained that the innovative multi-trait GS technology could be used to improve GN and other associated traits in rice breeding. Despite the concern regarding conventional agriculture, there is a profitable growing market share for high-quality vegetable products. In this view, Wilmer et al. compared the effects of fertilization with K_2_SO_4_ and KCl on the quality of two varieties of potatoes, an important staple crop, in a 2-year field experiment. Their evaluation of potato quality traits after harvest and after 5 months of storage at 6°C revealed that starch and ascorbic acid contents decreased, while soluble sugars and lipid-derived off-flavor compounds increased upon KCl supply, compared with K_2_SO_4_ supply; whereas minor effects were found on yield. Therefore, they showed that fertilization with KCl can strongly decrease potato quality. Finally, to meet the circular economy principles to transform agro-food residues into valuable economic resources, Ferri et al. proposed an optimized biotechnological approach to obtain peptides from a by-product of rice starch processing. They optimized the enzymatic hydrolysis parameters in small-scale bioreactors and fractionated the total digestates by cross-flow filtration and size exclusion chromatography. In this way, they found fractions with important antioxidant and anti-inflammatory properties that could be used as ingredients in food, nutraceutical, pharmaceutical, or cosmetic preparations. An in silico predictive analysis, based on the amino acid content was applied to estimate the taste properties of the fractions.

## Author contributions

The author confirms being the sole contributor of this work and has approved it for publication.
